# Improving Energy Efficiency in LoRaWAN Networks with Multiple Gateways

**DOI:** 10.3390/s23115315

**Published:** 2023-06-03

**Authors:** Ali Loubany, Samer Lahoud, Abed Ellatif Samhat, Melhem El Helou

**Affiliations:** 1 Ecole Supérieure d’Ingénieurs de Beyrouth (ESIB), Faculty of Engineering, Saint Joseph University of Beirut, Beirut 1107 2050, Lebanon; 2 Centre de Recherche Scientifique en Ingénierie (CRSI), Faculty of Engineering, Lebanese University, Beirut 1533, Lebanon

**Keywords:** Internet of Things, LPWAN, LoRaWAN, energy efficiency, spreading factor, power control

## Abstract

LoRaWAN has imposed itself as a promising and suitable technology for massive machine-type communications. With the acceleration of deployment, improving the energy efficiency of LoRaWAN networks has become paramount, especially with the limitations of throughput and battery resources. However, LoRaWAN suffers from the Aloha access scheme, which leads to a high probability of collision at large scales, especially in dense environments such as cities. In this paper, we propose EE-LoRa, an algorithm to improve the energy efficiency of LoRaWAN networks with multiple gateways via spreading factor selection and power control. We proceed in two steps, where we first optimize the energy efficiency of the network, defined as the ratio between the throughput and consumed energy. Solving this problem involves determining the optimal node distribution among different spreading factors. Then, in the second step, power control is applied to minimize the transmission power at nodes without jeopardizing the reliability of communications. The simulation results show that our proposed algorithm greatly improves the energy efficiency of LoRaWAN networks compared to legacy LoRaWAN and relevant state-of-the-art algorithms.

## 1. Introduction

Low-power wide area networks (LPWAN) address the stringent requirements of IoT applications where a massive number of low-cost, power-constrained nodes need to transmit small amounts of data over a long communication range [1]. LoRaWAN is one of the most widely used LPWAN technologies. Long Range (LoRa) is a wireless communication standard that operates in the unlicensed industrial, scientific, and medical (ISM) band using chirp spread spectrum (CSS) modulation developed by Semtech [2]. LoRaWAN is the medium access control MAC protocol standardized by the LoRa Alliance that runs on top of the LoRa physical layer. A LoRaWAN network has a star-of-stars architecture consisting of four entities: nodes, gateways, a network server, and an application server. Thus, there is no communication between nodes, and single-hop LoRa communication is used between nodes and gateways. The gateways simply forward the received messages to a central network server over an IP backbone. Precisely, there is no association between nodes and gateways, and a packet sent by a node can be received by multiple gateways. Moreover, the gateways also receive network server acknowledgments (ACK) or MAC commands and send them to the intended nodes. The central network server is in charge of managing network access and functionality, as well as routing messages between nodes and the LoRaWAN application.

The two main configurable parameters in the LoRa physical layer are the spreading factor (SF) and the transmission power (TP). SF refers to the number of bits encoded per symbol. This is the key parameter in LoRa because it affects the time of transmission, data rate, communication range, and power consumption. The greater the SF, the larger the coverage, and the lower the data rate. Lowering the data rate lengthens the transmission time and thus increases power consumption. The nodes closest to a gateway transmit with the lowest SF and highest data rate, thereby extending the battery life. More distant nodes transmit at a higher SF and require a longer time of transmission; therefore, their batteries will be drained quickly. Since SFs are quasi-orthogonal [3], communication with different SFs on the same frequency channel does not interfere with each other. As for the TPs, these are discrete values depending on the regional regulations [4]. Using higher TP values extends the communication range at the expense of increased power consumption.

The Aloha access scheme used in LoRaWAN increases the probability of collision and therefore reduces the network throughput, particularly when the density of nodes increases at some SFs. To overcome this problem, some nodes can be reconfigured to use higher SFs. However, this will lead to an increase in power consumption and degrade the energy efficiency of the network. Thus, energy efficiency can be improved by finding the best SF allocation and tuning the TPs of the nodes. In this paper, we introduce EE-LoRa, an algorithm that carefully distributes nodes among different SFs to enhance the energy efficiency of the LoRaWAN network and tunes TPs to the lowest possible levels.

The remainder of this paper is organized as follows. In Section 2, related work and our contributions are described. Section 3 presents an overview of LoRa and LoRaWAN technologies. Section 4 presents the system model. The EE-LoRa algorithm for SF selection and power control to improve the energy efficiency of LoRaWAN networks is described in Section 4. The simulation setup and obtained results are shown in Section 6. Finally, we conclude the article in Section 7.

## 2. Related Works and Contributions

In LoRa, a collision occurs when multiple nodes send simultaneous messages using the same SF over the same frequency channel. LoRaWAN is based on the Aloha access scheme, where nodes send traffic on a randomly chosen channel. When employing a large number of nodes, such a simple access protocol will increase the probability of collision drastically and therefore reduce the network throughput.

On the one hand, reconfiguring a portion of nodes to use higher SFs can improve the network throughput. On the other hand, this will increase the power consumption at nodes with limited batteries. Thus, improving the energy efficiency in such a network by optimizing the total throughput while minimizing the power consumption of battery-powered nodes at large scales is challenging. Moreover, nodes can save power by reducing the TP to the minimum level that ensures the reliability of communications. Consequently, the energy efficiency can be improved using a suitable algorithm that tunes both SF and TP to keep pace with the pervasive green trend in networking.

LoRaWAN introduced the adaptive data rate (ADR), a simple mechanism that aims to dynamically adjust the data rate at every node based on its link budget [5]. However, such an algorithm needs a long convergence time (hours to days). It is suitable only for stationary or slow-moving nodes with stable radio conditions [6]. In addition, it manages the transmission parameters at each node individually without considering the overall network performance. There are many recent studies for resource allocation to enhance the ADR scheme in dense LoRaWAN networks. A comparative study of the proposed solutions was presented in [7,8,9]. Some of them employ stochastic geometry, where SF selection is based on the distance between the node and the gateway [6,10,11]. However, such algorithms are limited to single-gateway scenarios. In addition, the link budget can differ for nodes the same distance from the gateway, especially in cities. Many other algorithms with different objectives were proposed to optimize LoRaWAN networks by configuring one or more LoRa resources (SF, TP, channel). A summary of recently proposed algorithms is given in Table 1.

The authors of [12] proposed an SF-selection algorithm that aims to maximize the minimum packet reception probability for all nodes considering energy consumption using a distributed genetic algorithm. An optimization problem for SF selection was devised in [13] in order to maximize the network throughput in multi-gateway scenarios. To achieve the optimal SF distribution, an adaptive algorithm that adjusts the signal-to-noise ratio (SNR) thresholds was developed. A heuristic algorithm for SF assignment named AD MAIORA was presented in [14]. The authors intended to decrease the load that some nodes put on some gateways by selecting a given SF. In multi-gateway scenarios, they iteratively found the best SF for each node. The authors of [15] proposed FADR, an SF-selection and TP-control algorithm for single-gateway deployment, to achieve an equal collision probability among different SFs while avoiding overly high TPs to reduce the energy consumption.

An MILP was formulated in [17] to derive the optimal SF and CF settings, considering the network traffic specifications, to reduce collisions and energy consumption in LoRaWAN networks. They evaluated the effectiveness of their approach in a single-gateway scenario. Compared to ADR, they improved the DER by 6% while reducing the network energy consumption.

The authors of [18] suggested a modified version of the MAC protocol LoRaWAN called LoRa+ for classes A and B consisting of receiving new channel parameters by nodes just before transmission. They showed that their approach decreased the packet error rate by up to 20% compared to legacy LoRaWAN when the number of nodes was less than 500.

The authors of [16] devise optimization problems to derive the optimal configuration at each node to improve reliability and minimize power consumption in dense LoRaWAN deployment. Since the proposed problems are nonlinear, they were transformed into integer linear programming (ILP) models and solved using IBM CPLEX. A two-stage optimization process was introduced to improve the delivery ratio and minimize the energy consumption in networks with multiple gateways. In the first stage, all nodes are assumed to use the highest TP. They proposed two approaches to assign SF to each node. The first, named OPT-MAX, aims to minimize the maximum probability of collision at each SF, like [20]. The second, named OPT-DELTA, derives the SF for all nodes in a way to balance the probability of collisions in all SFs. Once the SFs for all nodes are assigned, they apply the power-control stage, OPT-TP, to minimize the energy consumption of all nodes. They showed that their proposed solution outperforms the packet delivery ratio (PDR) by up to 8% compared to the state of the art with minimal energy consumption. However, the complexity of ILP is high and it requires a long computation time, which increases exponentially with the number of nodes.

The authors of [19] suggested a low-power multi-armed bandit (LP-MAB) mechanism, a centralized adaptive LoRaWAN configuration scheme. They intended to improve the PDR while reducing energy consumption. They used exponential weights for exploration and exploitation (EXP3), as well as the successive elimination (SE) methodology, to combine non-stationary adversarial and stochastic methods. They configured the transmission parameters of nodes centrally on the network server. The network server tries to learn the optimal set of transmission parameters for each node based on the reward, which is dependent on the receipt of acknowledgment messages.

The studies in [12,15,17] are limited to single-gateway deployments, whereas the studies in [12,13,14,17,18] lack a power-control strategy. In contrast to [19], whose method is focused on acknowledgment messages delivered from the network server, we focus our research on class A with unconfirmed transmission mode, where nodes do not request an acknowledgment from the network server after each transmission. Contrary to the work in [14,16], we do not consider the load of nodes at the gateway level, which requires more complicated calculations and a longer computation time. However, we concentrate on improving the overall network’s energy efficiency by extending its lifetime and increasing the throughput with our EE-LoRa algorithm. Moreover, after allocating the SFs and TPs of each node, we implicitly redistribute the load across the various gateways.

We summarize the major contributions of our work as follows:

- We propose a novel algorithm, named EE-LoRa, to improve the energy efficiency of LoRaWAN networks with multiple gateways. We devise an optimization problem that maximizes the energy efficiency of the network, defined as a benefit-cost ratio between the network throughput and the consumed energy.
- We use in our optimization problem a precise model of energy consumption that considers different modes of the class A LoRa transceiver over a period of time between two successive transmissions.
- We prove the convexity of our problem, and we use the Dinkelbach iterative method, where convergence is rapidly attained.
- Our algorithm, EE-LoRa, does not require signaling (beacons) or an additional synchronization process. It does not introduce any change to the LoRaWAN specification and can be applied to all LoRaWAN classes, particularly class A. Actually, the network server only sends downlink notifications to nodes that should change their configurable parameters (SF, TP) in one of their receiving windows.
- We evaluate our algorithm in a realistic scenario and compare the results to legacy LoRaWAN and relevant state-of-the-art algorithms, namely, OPT-MAX and OPT-DELTA. Moreover, we assess the results in different contexts related to minimum TP and idle current. We demonstrate that EE-LoRa outperforms the energy efficiency of legacy LoRaWAN and relevant state-of-the-art algorithms.

## 3. LoRa and LoRaWAN Overview

This section presents the aspects of LoRa and LoRaWAN technologies that are relevant to our work.

LoRa is a physical-layer technology developed by the Semtech corporation [2]. It modulates signals using a CSS technique that spreads a narrow-band signal over a wider channel bandwidth (BW). It supports multiple frequency channels (e.g., on the 433, 868, or 915 MHz ISM bands) depending on the region in which it is deployed [4]. This technique makes the signal robust to interference due to the processing gain of the spread-spectrum technique. As a result, the maximum power budget for LoRa operating in the 868 MHz band can exceed 150 dB (the receiver can decode transmissions 19.5 dB below the noise floor), thus enabling long communication ranges.

LoRa is characterized by various configurable parameters affecting the throughput, power consumption, and communication range, as detailed below.

- Carrier Frequency (CF): Represents the central frequency used in a band. LoRa operates in the unlicensed sub-GHZ ISM band, and the CF can be programmed within the range of 137 MHz to 1020 MHz in steps of 61 Hz. Specifically, the CF varies according to geographical regions, such as 868 MHz in Europe, 433 MHz in Asia, and 915 MHz in America. LoRa nodes typically support sixteen channels, while gateways usually support eight channels. In Europe, networks operate in the EU863–870 ISM band, which allows up to eight channels with a 125 kHz bandwidth each. Moreover, the three following channels (868.1, 868.3, and 868.5 MHz) are mandatory [4].
- Spreading Factor (SF): Refers to the number of bits encoded per symbol. This is the key parameter in LoRa because it affects the time of transmission, data rate, communication range, and power consumption. LoRa supports multiple SFs ranging from seven to 12. Table 2 gives the variation of the data rate, sensitivity, and SNR thresholds of different SFs for the 868 MHz band. Note that for SNR values lower than −20 dB, a node is considered out of network coverage. Going from SF 7 to SF 12 decreases the receiver’s sensitivity from −123 dBm to −137 dBm and, therefore, extends the coverage. However, this comes at the expense of lowering the data rate and increasing the transmission time and energy consumption. The throughput limitation in LoRa can be seen from Table 2, where the data rate varies between 0.3 (using the highest SF 12) and 5.4 kbps (using the lowest SF 7). We note also that the different SFs are quasi-orthogonal [3], which enables the simultaneous reception of packets with different SFs.
- Bandwidth (BW): Defines the spectrum occupied by a symbol. It can be set to 125, 250, or 500 kHz. A larger bandwidth corresponds to higher data, at the expense of reducing sensitivity.
- Coding Rate (CR): LoRa uses a forward error correction (FEC) scheme to perform error detection and correction by adding redundancy bits, improving the robustness of the transmitted signal. The coding rate can be set to $\frac{4}{4+CR}$ with CR values ranging from 1 to 4. Increasing the CR value gives more protection against interference. However, this will increase the transmission time and power consumption.
- Transmission Power (TP): It can be adjusted from −4 dBm to 20 dBm in 1 dB steps. Due to hardware implementation limitations, the range frequently varies from 2 dBm to 20 dBm. The highest TP is subject to regional regularity constraints, e.g., 14 dBm in Europe [4]. Using higher TP values extends the communication range at the expense of increased power consumption.

**Table 2:** Data rate, sensitivity and SNR thresholds of different SFs for the 868 MHz band, bandwidth 125 kHz, coding rate 4/5 [21].

SF	Data Rate [kbps]	Sensitivity [dBm]	Required SNR [dB]
7	5.458	−123	−7.5
8	3.125	−126	−10
9	1.757	−129	−12.5
10	0.976	−132	−15
11	0.537	−134.5	−17.5
12	0.293	−137	−20

A LoRa symbol (chirp) is made up of $2^{SF}$ chips in which the chip rate equals the BW. For example, SF 9 means that each chirp consists of $2^{9}$ RF chips carrying nine data bits. The symbol duration can be expressed according to the following formula [2]:

(1) $$T_{sym}=\frac{2^{SF}}{BW}$$

Thus, the symbol rate $R_{s}$ (symbols/s) can be deduced as follows:

(2) $$R_{s}=\frac{1}{T_{sym}}=\frac{BW}{2^{SF}}$$

Having a code rate CR, we can express the data rate ($R_{b}$) in bits/s as follows [20]:

(3) $$R_{b}=SF\times \frac{BW}{2^{SF}}\times \frac{4}{4+CR}$$

**Figure 1:**
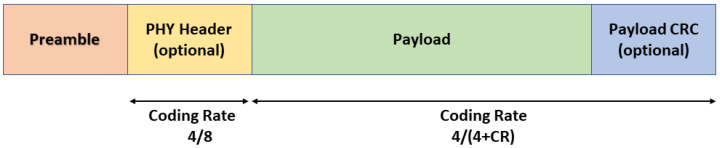
LoRa frame structure.

The frame structure at the physical layer comprises a preamble, an optional header, and the data payload, as shown in Figure 1. The preamble allows the receiver to be synchronized with the transmitter. The preamble contains a number of $n_{preamble}$ up chirp symbols with 4.25 LoRa symbols as frame delimiters for synchronization. As a result, the preamble length is equal to ($n_{preamble}+4.25$) symbols. Thus, the preamble duration can be expressed as follows:

(4) $$T_{preamble}=\left(n_{preamble}+4.25\right)\times T_{sym}$$

Because the preamble size scales with SF and there is no single preamble for all SFs, the transmitter and receiver must know the SF in advance in order to detect the preamble. There is an optional PHY header following the preamble. This header is transmitted with the highest code rate of 4/8 when it is present. The remaining portion of the frame is encoded using the code rate specified in the PHY header. The payload is sent after the header. The maximum payload size is defined for each region in the LoRaWAN Regional Parameters [4]. For the EU863-870MHz band, the maximum payload size varies between 51 bytes for the slowest data rate (SF 12, BW 125 kHz) and 222 bytes for the faster rate (SF 7, BW 125 kHz). There is an optional payload CRC at the end of the frame.

The number of symbols $n_{payload}$ that comprise the header and the packet payload is calculated with Equation (5). We denote by PL the size of the payload in bytes; *H* is equal to 0 when the header is enabled and 1 otherwise; and DE=1 if low-data-rate optimization is enabled (SF≥11), and 0 otherwise [2].

(5) $$n_{payload}=8+max\left(ceil\left(\frac{8PL-4SF+28+16-20H}{4(SF-2DE)}\right)\left(CR+4\right),0\right)$$

Then, we can deduce the payload duration as follows:

(6) $$T_{payload}=n_{payload}\cdot T_{sym}$$

The time on air (ToA) of a LoRa transmission is computed according to the payload size and depends on several parameters: SF, BW, and CR. These parameters can cause significant variation in the transmission time. The time on air is simply obtained as the sum of the preamble and payload duration: for a given payload size, and at a given BW and coding rate, we denote by ToA(s) the time to transmit a packet at SF *s*. Table 3 presents the ToA configuration assuming a 125 kHz bandwidth and a coding rate of 4/5. It can be seen that the ToA increases when the SF is incremented.

On top of the physical layer runs the medium access control MAC protocol, LoRaWAN, standardized by the LoRa Alliance [5]. This open-access standard describes the network architecture and defines the communication protocol used to connect each network entity. LoRaWAN is designed as a single-hop star network. While this greatly simplifies the deployment and operation, it also implies that enough gateways should be available to cover the deployment area.

**Figure 2:**
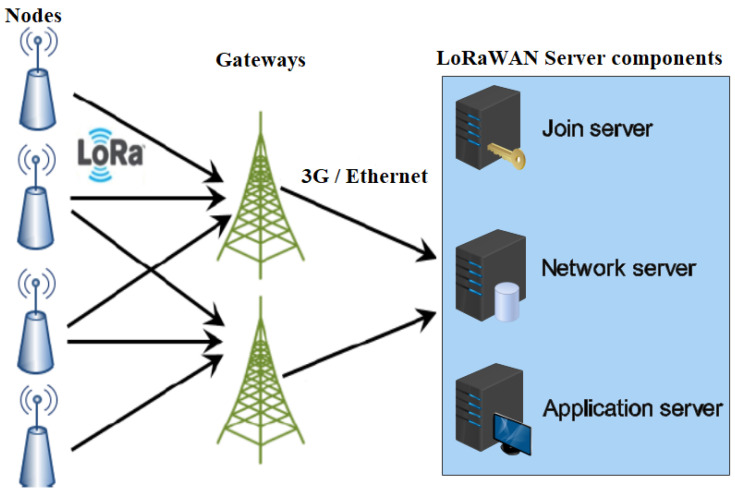
LoRaWAN architecture.

A LoRaWAN network has a star-of-stars topology, as illustrated in Figure 2. It is made up of the following entities [5]:

- Nodes: They are also known as end-devices or motes. Each node contains basically a microcontroller, a radio unit, and other peripherals such as sensors, and only transmits data to gateways using single-hop LoRa communication.
- Gateways: They are made up of a LoRa transceiver chip and a baseband processor. They can decode multiple channels simultaneously and support between eight and 64 channels. They simply relay received messages to a central network server via an IP backbone. The IP traffic from a gateway to the network server can be via Wi-Fi, hardwired Ethernet, or a cellular connection. The packet forwarder specifies the protocol used to communicate with the network server. There are various packet forwarders; the two most popular ones are the Semtech User Datagram Protocol (UDP) and Message Queuing Telemetry Transport (MQTT) [22]. The majority of LoRa gateways still come pre-compiled with the Semtech UDP forwarder, which was the original packet forwarder. Gateways also receive network server acknowledgments (ACK) or MAC commands and send them to the intended nodes. Note that there is no association between nodes and gateways, and a packet sent by a node can be received by multiple gateways.
- LoRaWAN Server: Consists of a central network server, an application server, and a join server. The LoRAWAN specification treats these servers as if they are always co-located. The network server is the center of the star architecture. It manages network access and functionality and is responsible for routing messages between nodes and LoRaWAN application. It handles node authentication and authorization, data transmission, data rate adaptation, and duplicate packet elimination. The application server processes all data messages received from nodes. Moreover, it generates and sends all application-layer downlink payloads to connected nodes via the network server. Several applications can be connected to a single network server. The join server is in charge of managing the over-the-air (OTA) node-activation procedure. It sends the node’s network session key to the network server and the application session key to the appropriate application server.

When a node sends a message to the gateway, it is referred to as an uplink, whereas when the gateway sends a data packet to the node, it is known as a downlink. Furthermore, there are two types of messages in any LoRaWAN operation: (1) unconfirmed messages, in which the nodes do not request a response from the network server, and (2) confirmed messages, in which the nodes do request a response from the network serer.

**Figure 3:**
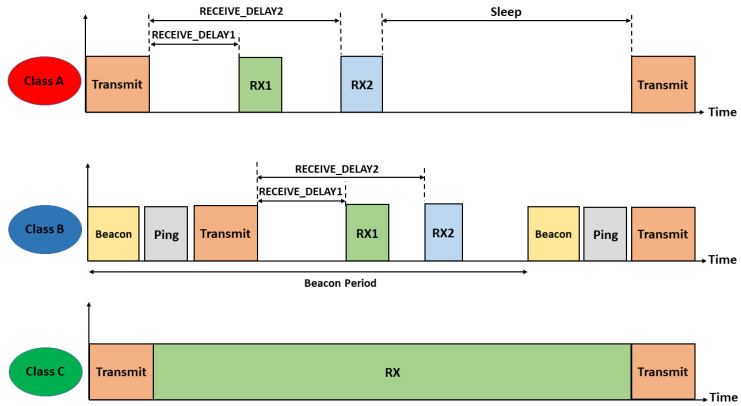
Time diagram of different LoRaWAN classes.

LoRaWAN defines three different classes of nodes with different capabilities and power requirements, as illustrated in Figure 3. Class A supports basic bidirectional communications; two receive windows RX1 and RX2 will be opened after RECEIVE_DELAY1 and RECEIVE_DELAY2, respectively, from transmitting uplink messages. During these two receive windows, the network server can send acknowledgment and MAC commands to nodes so as to control transmission parameters such as the spreading factor, power, and bandwidth. RX1 uses the same SF as the original uplink, while RX2 uses SF 12 and is opened only if the downlink message is not received during RX1. Class A implementation is required for all devices when nodes send sporadic data to gateways through an Aloha-based access mechanism. Class A nodes spend most of their time in the sleep state and consume the least energy. Class B extends Class A by adding extra receive windows at scheduled times. The nodes are synchronized by the periodic broadcast of beacons from gateways. Finally, nodes of Class C are permanently listening to the channel with continuously open reception windows.

## 4. System Model

### 4.1. Network Model

**Figure 4:**
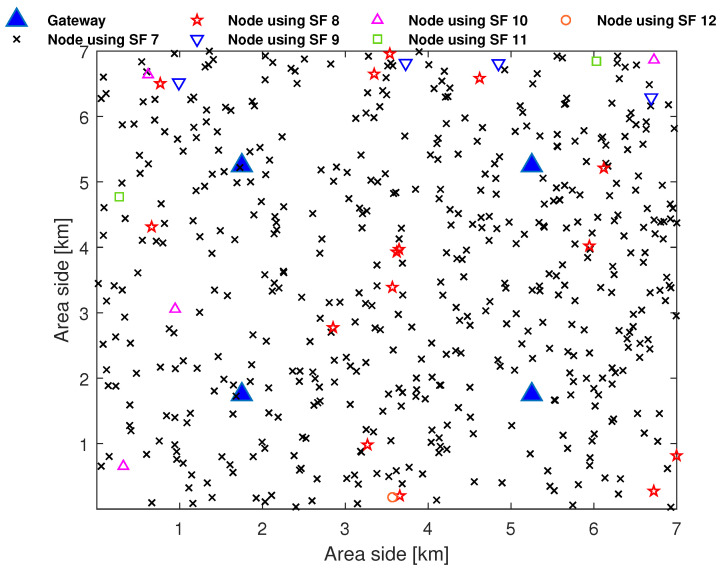
Example of distribution of 500 nodes in a 4-gateway LoRaWAN network.

Let us consider a LoRaWAN network with *r* gateways regularly distributed in an urban square area and an average of $N_{t}$ nodes distributed following a Poisson point process. Let $f_{1}$ be the fraction of nodes located indoors and $f_{2}=1-f_{1}$ be the fraction of nodes located outdoors. Table 4 lists the required SNR to correctly decode messages at different SFs [23]. Figure 4 illustrates the distribution of $N_{t}=500$ nodes among different SFs in a 4-gateway LoRaWAN deployment in a square area with a side length of 7 km.

Table 5 lists numerical results related to Figure 4. Each node in this example uses the maximum allowed TP (14 dBm) and selects the lowest SF, according to Table 4, given its own radio conditions. Note that if a node is covered by multiple gateways, it will be assigned the lowest SF based on the highest SNR received by one of the gateways. We denote by D(s) the number of nodes selecting the SF *s* and by N(s) the number of nodes that use SF ≥ *s*.

**Figure 5:**
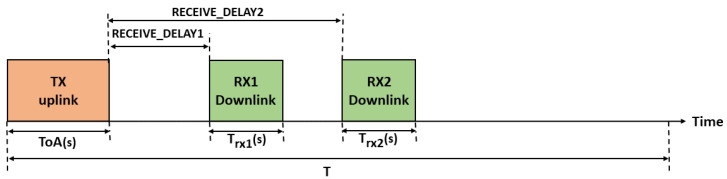
Time diagram for a class A node during a period of time *T*.

We assume that all nodes are configured with the same modem settings (bandwidth, coding rate, preamble length, etc.) and transmit packets on a single channel with the same payload size *k*. Therefore, the time on air to transmit a packet depends only on the used SF and is denoted by ToA(s). A detailed calculation of the time on air is given in [2]. Transmit attempts for all nodes are performed according to a Poisson distribution with a mean arrival rate of λ packets per second. Then, given a duty cycle limitation of *d* = 1%, the packet generation rate for each node using SF *s* must verify λ.ToA(s)≤d. We suppose that all nodes in the network are of class A with unconfirmed transmission mode [5]; then, each node opens two receive windows RX1 and RX2 after RECEIVE_DELAY1 and RECEIVE_DELAY2 from the transmitting uplink message even if no acknowledgment is expected, as shown in Figure 5. These two receive windows are the only opportunity for the network server to send notifications and new setup to nodes. Note that RX2 is only opened if neither data nor acknowledgment is received during RX1. R_D1 and R_D2 refer to RECEIVE_DELAY1 and RECEIVE_DELAY2, respectively. Let *T* = 1/λ be the time period between two successive uplink transmissions, and let $T_{rx1}\left(s\right)$ and $T_{rx2}\left(s\right)$ be the first and second receive window durations, respectively. Table 6 lists various variables and parameters used in our model.

### 4.2. Network Utility Model

In this section, we will present the network utility function and study its convexity. LoRaWAN uses the pure Aloha random access mechanism, where nodes transmit packets without channel sensing. Then, the Aloha mathematical equations can be used to estimate the expected performance of a typical LoRaWAN deployment [24]. We denote by p(s) the ratio of nodes that use SF s∈Ω={7,8,9,10,11,12}. $S_{min}$ refers to the minimum SF 7 and $S_{max}$ to the maximum SF 12.

Assuming that the transmit attempts are performed according to a Poisson distribution with a mean arrival rate of λ packets per second, the normalized channel traffic load on each SF *s* can be calculated as follows [25]:

(7) $$\begin{array}{cc} G(s) & =\lambda .D(s).ToA(s)\\ \; & =\lambda .p\left(s\right).N_{t}.ToA\left(s\right) \end{array}$$

The probability of having *k* transmissions during two packet times is expressed according to the Poisson distribution, as follows [25]:

(8) $$P\left(k\;\mathrm{transmissions}\right)=\frac{{\left(2G\right)}^{k}\exp\left(-2G\right)}{k!}.$$

Then, the probability of a successful frame transmission (k=0) is equal to $e^{-2.G(s)}$. The Aloha throughput per frame time can be obtained as the normalized channel load multiplied by the success probability: $G\left(s\right).e^{-2.G(s)}$. Thus, to obtain the throughput (S) in bits/s on each SF, we divide by the frame time and multiply by the packet length. S is expressed as follows [26]:

(9) $$\begin{array}{cc} S(s) & =\lambda .D\left(s\right).b.e^{-2.G(s)}\\ \; & =\lambda .p\left(s\right).N_{t}.b.e^{-2.\lambda .p\left(s\right).N_{t}.ToA\left(s\right)} \end{array}$$

where *b* represents the number of bits per packet and is equal to the sum of the payload size *k* and the preamble length $n_{pream}$.

We define the utility function of the network as the total throughput (R) of the LoRaWAN network in bits/s. As the different SFs are assumed to be orthogonal, R can be calculated as the sum of the throughput on each SF:

(10) $$R=\sum_{s=S_{min}}^{S_{max}}S\left(s\right)$$

### 4.3. Energy Consumption Model

**Figure 6:**
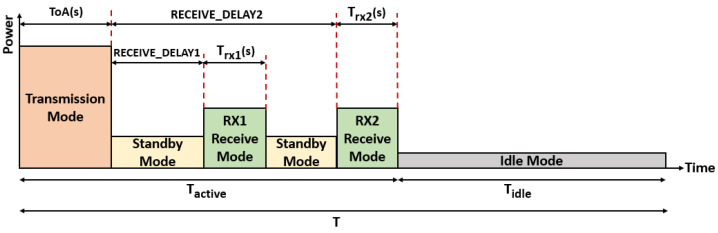
Transceiver modes during a period of time T.

In our study, we focused on the power consumption at the transceiver. We assume that all nodes use the same LoRa transceiver, SX1272 [23]. At each period *T*, we consider four transceiver modes: transmission, standby, receive, and idle. After transmitting an uplink message, one of the following situations will occur:

- $S_{1}$: The node successfully receives a downlink message at RX1 with probability $d_{1}$; then, it will switch to idle mode.
- $S_{2}$: The node does not receive a downlink message during RX1 with probability $d_{2}=1-d_{1}$, as shown in Figure 6.

The current consumptions $I_{st}$, $I_{rx}$, and $I_{id}$ in the standby, receive, and idle modes, respectively, are obtained from [23]. The consumed current at transmission $I_{tx}\left(l\right)$ depends on the power level *l*. The amount of energy consumed by the nodes using SF *s* during the active period $T_{active}$, which is the time before the transceiver enters idle mode, can then be expressed as follows:

(11) $$\begin{array}{cc} E_{active}\left(s\right) & =d_{1}.p\left(s\right).N_{t}.V.\left[ToA\left(s\right).I_{tx}\left(l\right)+R\_D1.I_{st}+T_{rx1}\left(s\right).I_{rx}\right]\\ \; & \;+d_{2}.p\left(s\right).N_{t}.V.[ToA\left(s\right).I_{tx}\left(l\right)+\left(R\_D2-T_{rx1}\left(s\right)\right).I_{st}\\ \; & \;+\left(T_{rx1}\left(s\right)+T_{rx2}\left(s\right)\right).I_{rx}] \end{array}$$

The energy consumed by nodes using SF *s* during idle period $T_{idle}$ is defined by:

(12) $$\begin{array}{cc} E_{idle}\left(s\right) & =d_{1}.p\left(s\right).N_{t}.V.\left[\left(T-\left(ToA\left(s\right)+T_{rx1}\left(s\right)+R\_D1\right)\right).I_{id}\right]\\ \; & \;+d_{2}.p\left(s\right).N_{t}.V.\left[\left(T-\left(ToA\left(s\right)+R\_D2+T_{rx2}\left(s\right)\right)\right).I_{id}\right] \end{array}$$

Thus, the energy consumption on each SF *s* is obtained by:

(13) $$E\left(s\right)=E_{active}\left(s\right)+E_{idle}\left(s\right)$$

The total consumed energy (E) of the LoRaWAN network during period *T* is hence computed as the sum of this consumed energy on each SF:

(14) $$E=\sum_{s=S_{min}}^{S_{max}}E\left(s\right)$$

We define ($E_{active}$) as the total consumed energy during active period $T_{active}$ and it is calculated as the sum of $E_{active}$ on each SF:

(15) $$E_{active}=\sum_{s=S_{min}}^{S_{max}}E_{active}\left(s\right)$$

## 5. EE-LoRa Algorithm

In this section, we will present our algorithm, named EE-LoRa, that derives the best SF and TP for each node in a way to improve the energy efficiency of LoRaWAN networks. First, we compute the optimal node distribution among different SFs in Section 5.1. Then, we adjust the SNR thresholds in Section 5.2 to satisfy the obtained optimal node distribution. Next, a power-control step is applied in Section 5.3. Finally, thee nodes will be notified about their new configuration as described in Section 5.4.

### 5.1. Optimization Problem for SF Selection

#### 5.1.1. Problem Formulation

At this step, we suppose that all nodes use the maximum allowed TP (14 dBm as per the regional regulation). We formulate the optimization problem (P) of SF selection in order to maximize the network’s energy efficiency (Eff), defined as the ratio of the network utility function (R) to the total consumed energy (E) during period *T* [27]. This is a multi-objective problem in which the optimal node distribution for each SF is determined and their ratios are computed.

The objective function can be expressed as follows:

(16) $$\left(P\right):\max_{p(s)}Eff=\max_{p(s)}\frac{R}{E}$$

subject to

(17) $$\sum_{s=S_{min}}^{S_{max}}p\left(s\right)\leq 1$$

(18) $$\sum_{i=s}^{S_{max}}p\left(i\right)\geq \frac{N(s)}{N_{t}},\forall s\in \Omega$$

Constraint (17) ensures that the sum of ratios does not exceed 1. Meanwhile, constraints (18) limit the number of nodes selecting SF *s* and higher to the maximum number N(s), since a node using SF *s* cannot select an SF lower than *s*. This fractional problem is non-convex, so it cannot be solved directly. We adopt the Dinkelbach method, which transforms (P) into a parameterized convex programming problem as follows [28]:

(19) $$\begin{array}{cc} \; & \left(\hat{P}\right):F\left(\eta \right)=\max_{p(s)}\left(R-\eta E\right)=\min_{p(s)}\left(-\left(R-\eta E\right)\right) \end{array}$$

subject to:

(20) $$\sum_{s=S_{min}}^{S_{max}}p\left(s\right)\leq 1$$

(21) $$\sum_{i=s}^{S_{max}}p\left(i\right)\geq \frac{N(s)}{N_{t}},\forall s\in \Omega$$

where η is a non-negative parameter.

#### 5.1.2. Convexity Study

The constraints are linear and (E) is affine. However, we need to study the convexity of (R). Thus, we study the convexity of throughput S(s) at each SF *s* that depends only on the number of nodes D(s). The second derivative of S is given by $4\lambda^{2}.b.ToA\left(s\right).\left(\lambda .D\left(s\right).ToA\left(s\right)-1\right).e^{-2.\lambda .D(s).ToA(s)}$. Hence, the condition to guarantee the concavity of S is expressed by:

(22) λ.D(s).ToA(s)<1

Thus, the number of nodes D(s) at each SF *s* should verify this condition:

(23) $$D\left(s\right)<\frac{1}{\lambda .ToA(s)}$$

For example, with the same packet generation rate λ=6 packets/h and the same packet length *k* of 40 bytes for all nodes, we can deduce the maximum number of nodes at each SF to ensure the convexity of the optimization problem, as shown in Table 7. A network with 12,614 nodes with up to 5612 of them selecting SF 7 still works in the convexity domain. Therefore, the region that ensures the convexity of our optimization problem is large and sufficient in a realistic network.

We will satisfy condition (23) in our study to guarantee that R is concave, and therefore ensure the convexity of the optimization problem.

#### 5.1.3. Problem Solution

We denote by $\eta^{*}$ the optimal solution of the original problem (P). It can be expressed by:

(24) $$\begin{array}{cc} \eta^{*} & =\max_{p(s)}\left(\frac{R}{E}\right)\\ \; & =\frac{R(p^{*}\left(s\right))}{E(p^{*}\left(s\right))} \end{array}$$

$\eta^{*}$ is the maximum of the problem (P) if and only if:

(25) $$\begin{array}{cc} F(\eta^{*}) & =\max_{p(s)}\left(R-\eta^{*}E\right)\\ \; & =0 \end{array}$$

F(η) is continuous and strictly decreasing in η. Then, we can derive its unique root $\eta^{*}$ using the Dinkelbach iterative algorithm, summarized in Algorithm 1. At each iteration, we solve the inner problem $\left(\hat{P}\right)$ (Line 3) using the nonlinear programming solver provided in MATLAB fmincon. It is a gradient-based method that is designed to work on problems where the objective and constraint functions are both continuous and have continuous first derivatives. Then, we update the η value in (Line 6) until $F^{n}$ becomes ≤ϵ, where ϵ represents a maximum given tolerance. Note that the obtained local optimum will be considered global since we work in the concavity domain of R.
**Algorithm 1:** Dinkelbach algorithm **Require:** Initial value $\eta^{0}$, maximum tolerance ϵ≥0, iteration n←0  1: **do**  2:  $\eta \leftarrow \eta^{*}$  3:  Solve $\left(\hat{P}\right)$ in ( 19)  4:  5:  $p^{n}\leftarrow$ arg $\max_{p(s)}\left(R-\eta E\right)$  6:  $F^{\left(n\right)}\leftarrow$$\max_{p(s)}\left(R-\eta E\right)$  7:  8:  $\eta^{n+1}\leftarrow$$\frac{R(p^{n})}{E(p^{n})}$  9:  n←n+1  10:  **while** $F^{\left(n\right)}>\epsilon$

### 5.2. SNR Threshold Adjustment

The required SNR values necessary to demodulate LoRa signals at different SFs are given in Table 4. For example, a node should have an SNR value between −10 and −7.5 dB to be assigned SF 8, and each node with an SNR value less than −20 dB is considered uncovered. In our work, we adjust the required SNR thresholds based on the obtained optimal node distribution. We apply the adaptive SNR threshold method presented in [13] to obtain the new SNR threshold values for each SF *s*, denoted by computed_SNR_thr(s).

**Figure 7:**
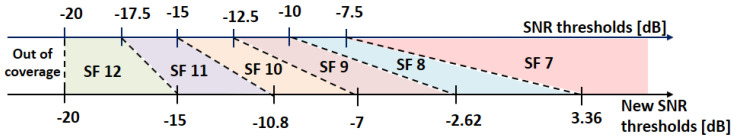
Example of adjusting the SNR thresholds.

First, the network server arranges the received SNR from all nodes in descending order. Next, we deduce the optimal number of nodes at each SF from the calculated optimal ratios p(s). Then, we find the computed_SNR_thr(s) by adjusting the SNR thresholds at each SF *s* to the lowest SNR value in the corresponding optimal set of nodes. Finally, each node will be assigned a new SF based on the computed_SNR_thr(s). An illustrative example of this process is shown in Figure 7. For example, if the SNR of a node is −2 dB, it is assigned SF 8 instead of SF 7, following the computed_SNR_thr(s).

### 5.3. Power Control

The current consumption at transmission $I_{tx}$ depends on the TP level *l*. Table 8 gives $I_{tx}$ when *l* varies between 2 and 14 dBm for a SX1272 LoRa transceiver obtained from the Semtech LoRa Calculator [29]. For instance, decreasing the TP level from 14 dBm to 2 dBm will mitigate $I_{tx}$ from 44 to 24 mA. As a result, in order to minimize the energy consumption, we will reduce the TP for all nodes to the bare minimum required to ensure communication reliability.

We consider in our work two different sets of TP levels that differ by TP step, listed in Table 9. The TP step of 3 dB used in the first set $T_{1}$ is the same applied in the ADR mechanism introduced by LoRaWAN [6]. However, we decrease the TP step in the second set $T_{2}$ to 1 dB. For example, having a TP level variation between 2 and 14 dBm for Sa X1272 LoRa transceiver, $T_{1}$ is constituted by 5 levels while $T_{2}$ is composed of 13 TP levels. These two sets are used to evaluate the impact of increasing the number of TP levels later.

After finding the computed_SNR_thr(s) at each SF and deducing the SF *s* for each node in the network as detailed in Section 5.1, and, having its SNR and initial TP denoted by old_TP, we can calculate its new TP denoted by new_TP as follows:

(26) $$\begin{array}{cc} new\_TP & =computed\_SNR\_thr(s)-SNR+old\_TP \end{array}$$

Since TP levels are discrete [23], the obtained TP value for each node is rounded to the nearest highest level in the used set of TPs.

### 5.4. Notification

After computing the optimal SF and TP for each node in the network, the network server only needs to send downlink notifications to the nodes that have had changes in their radio conditions. Each class A node will be notified about its new configuration, either in RX1 or RX2, using the DataRate_TXPower byte of the LinkADRReq command. Note that EE-LoRa does not involve radical changes to the LoRaWAN specifications.

## 6. Performance Evaluation

### 6.1. Simulation Setup

To represent a multi-gateway LoRaWAN network in a dense environment, we consider a 4-gateway deployment in an urban square region with a 7 km side length and $N_{t}$ nodes, where $N_{t}$ is a Poisson-distributed random variable with mean $\bar{N_{t}}$. Various scenarios are considered in which $\bar{N_{t}}$ ranges between 1000 and 4000. To imitate a realistic model, the Okumura–Hata path-loss model for the urban environment is considered, where the fraction of nodes that are located indoors (dense urban) $f_{1}=\frac{1}{2}$. The remaining fraction, $f_{2}$, is made up of outdoor nodes. Log-normal shadowing with a zero mean and 8 dB standard deviation is assumed. We assume that all nodes are of class A with unconfirmed transmission and generate the same traffic intensity λ. Since we have a massive number of nodes, we suppose that the two situations $S_{1}$ and $S_{2}$ that may occur after transmitting an uplink message are equiprobable, like the assumption taken in [30]. Consequently, $d_{1}=d_{2}=\frac{1}{2}$. Considering a payload length of *k* = 40 bytes, we set λ to the minimum packet generation rate of 18 packets/h. It corresponds to the highest SF 12 and the longest time on air ToA value as shown in Table 10.

In our work, a single channel from the three default channels in the EU863–870 ISM band with a bandwidth of 125 kHz and duty cycle *d* of 1% is considered, without loss of generality. Therefore, we divide the traffic intensity by 3. Each node sends an uplink message periodically at a packet generation rate of 6 packets/h. Such data rate and packet generation rate are suitable for smart buildings, waste management, and agriculture monitoring applications where we need to transmit small amounts of data at low data rates.

The simulation parameters are listed in Table 11. Note that, in the simulation, all nodes are always covered. The simulation is performed using MATLAB, and the numerical results are obtained from 30 runs. We use a random starting point for fmincon and we consider the local minimum as global since we work in the domain that ensures the convexity of our optimization problem. Table 12 represents an example of 4000-node distribution among different SFs before using our algorithm according to the required SNR at each SF. This node distribution satisfies the convexity condition of our optimization problem.

The following metrics are used in the performance evaluation:

- The total throughput is computed as in Equation (10);
- The total consumed network energy during *T* is computed as in Equation (14);
- The overall energy efficiency Eff of the LoRaWAN network represents the objective of our optimization problem (P);
- The LoRaWAN network’s active energy efficiency is defined as the ratio of the total network throughput to the total network consumed energy during period $T_{active}$, expressed as follows:
  (27) $$Eff_{active}=\frac{R}{E_{active}}$$

### 6.2. Simulation Results

#### 6.2.1. Algorithm Evaluation

We first evaluate EE-LoRa for SF selection and power control with $T_{1}$ TP levels. We assume that all nodes are static. The number of nodes varies between 1000 and 4000. We compare the obtained results to legacy LoRaWAN and the appropriate algorithms listed below:

- Legacy LoRaWAN: Each node is assigned the lowest SF that allows it to reach the nearest gateway using the highest TP (14 dBm), according to Table 4. To make a fair comparison, the TP for each node is then reduced to the lowest value, which permits it to reach a gateway with the selected SF.
- OPT-MAX [16]: Minimizes the highest probability of collisions in any given SF. Then, the TP for each node is derived using the integer linear programming algorithm OPT-TP [16], which aims to minimize the network energy consumption.
- OPT-DELTA [16]: Derives the SF for all nodes in a way to balance the probability of collisions in all SFs. After that, the optimal TPs for nodes are obtained using OPT-TP.

**Figure 8:**
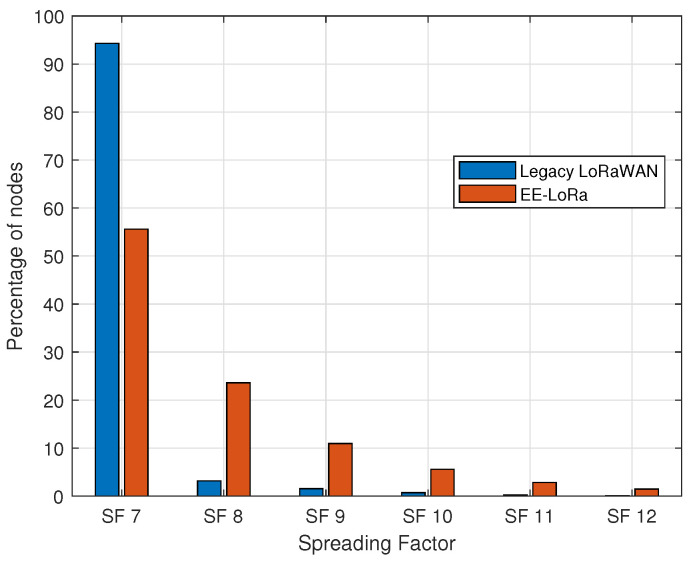
Percentage of nodes over different SFs when $N_{t}=4000$.

Figure 8 illustrates the node distribution over different SFs obtained using EE-LoRa compared to legacy LoRaWAN when the number of nodes is 4000. The percentage of nodes at SF 7 exceeds 94% when using legacy LoRaWAN. Meanwhile, the distribution of nodes over other SFs is below 6%. The highest percentage of nodes at SF 7 can be explained by the best coverage and highest received SNR in multi-gateway deployment. However, such a distribution dramatically increases the number of collisions and reduces the network throughput since LoRaWAN is based on the Aloha protocol. On the other hand, it can be seen that our proposed algorithm redistributes the nodes over different SFs, which reduces the collision probability and improves the network throughput.

**Figure 9:**
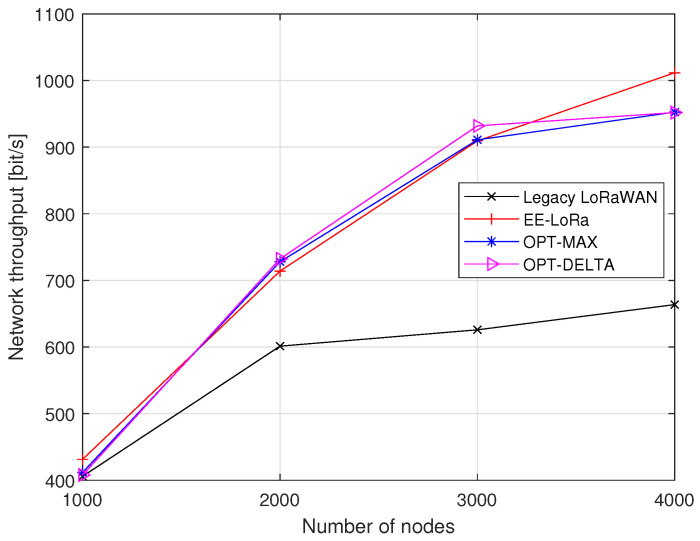
Network throughput comparison.

Figure 9 represents the obtained total throughput of the LoRaWAN network. EE-LoRa always outperforms the total throughput of the legacy LoRaWAN, especially when the number of nodes increases. The obtained network throughput using OPT-MAX and OPT-DELTA are very close to that acquired using EE-LoRa when the number of nodes is lower than 3000. When the number of nodes is 4000, the total throughput improvement of EE-LoRa achieves 53%, 6.2%, and 6.28% compared to legacy LoRaWAN, OPT-MAX, and OPT-DELTA, respectively.

**Figure 10:**
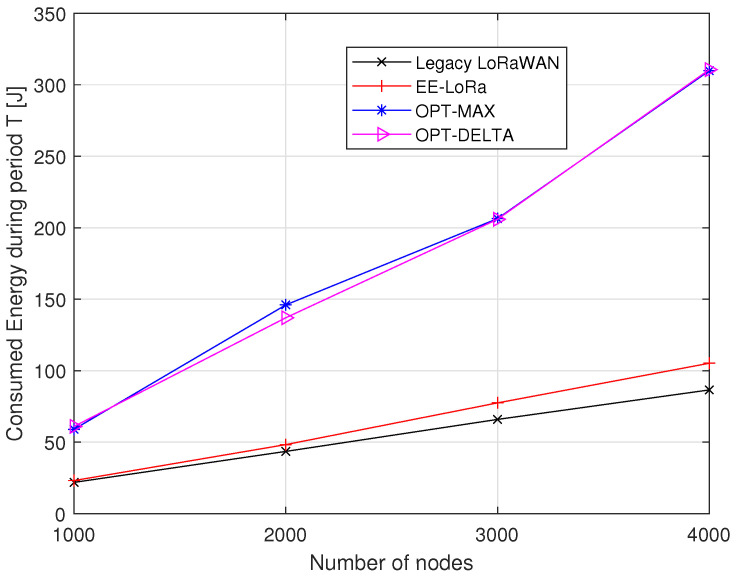
Comparison of energy consumption during period *T*.

Figure 10 depicts the LoRaWAN network’s energy consumption during period *T*, which represents the time between two consecutive transmissions. We can show that EE-LoRa consumes more energy than legacy LoRaWAN, in which each node chooses the lowest SF without regard for network performance. However, the energy consumption using OPT-MAX and OPT-DELTA are very high compared to EE-LoRa. For instance, when the number of nodes is 4000, the energy consumed using OPT-MAX and OPT-DELTA is three times higher than that obtained using EE-LoRa.

**Figure 11:**
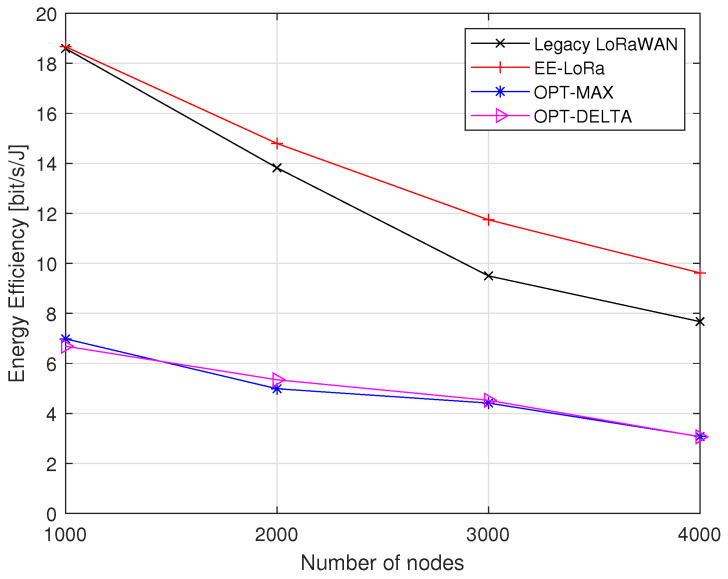
Energy efficiency comparison.

The results in Figure 11 show the comparison in terms of energy efficiency. The energy efficiency of the network decreases as the number of nodes increases, but EE-LoRa always achieves the highest energy efficiency. When the number of nodes reaches 4000, the energy efficiency improvement of EE-LoRa becomes about 25%, 221%, and 222% compared to legacy LoRaWAN, OPT-MAX, and OPT-DELTA, respectively. To obtain a better improvement, we can increase the number of TP levels and reduce the idle current, as discussed hereafter.

**Figure 12:**
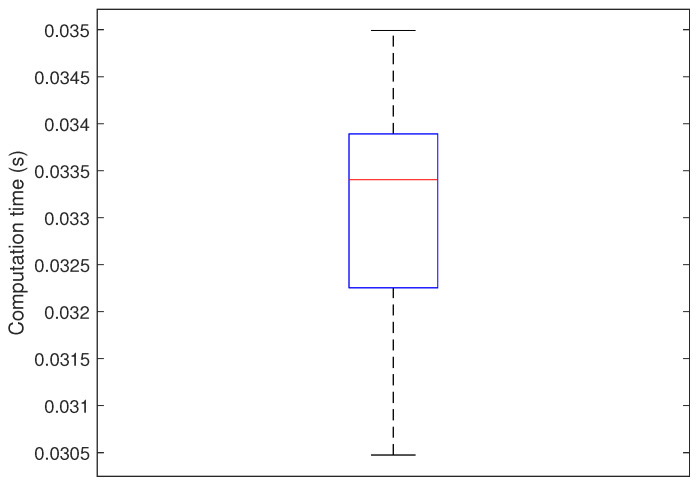
Computation time to solve our optimization problem when $N_{t}$ = 4000 nodes.

The complexity performance in our optimization problem does not depend on the number of nodes. In our proposed multi-objective problem, we find the optimal node distribution for each SF by computing their ratios p(s). Figure 12 illustrates the boxplot of the computation time to solve our proposed optimization problem from 30 simulation runs when the number of nodes is 4000 nodes. The computation time is evaluated on a 2.6 GHz intel i7 computer with 16 GB of memory. We can show that the convergence time of our optimization approach does not exceed 0.035 s, which is a fast computation time. In contrast, the complexity performance of ILP optimization used in OPT-MAX and OPT-DELTA is high and requires a long computation time, which increases exponentially with the number of nodes. With the minimum number of nodes 1000, the execution time of these algorithms (OPT-MAX and OPT-DELTA) exceeds 12 s, which is very high compared to the execution time of our algorithm.

#### 6.2.2. Impact of Increasing the Number of TP Levels

We evaluate EE-LoRa in terms of overall energy efficiency and active energy efficiency as defined by Equation (27) with 4000 nodes in two different scenarios using two TP sets of TP $T_{1}$ and $T_{2}$, which differ by the TP step, 1 dB and 3 dB, respectively. The maximum allowed TP level is constant and equals 14 dBm, and we change the minimum TP. For example, when the minimum TP is 8 dBm, we will have 3 TP levels (14, 11, and 8 dBm) for $T_{1}$ and 7 TP levels (14, 13, 12, 11, 10, 9, and 8 dBm) when using $T_{2}$ at the power-control step.

**Figure 13:**
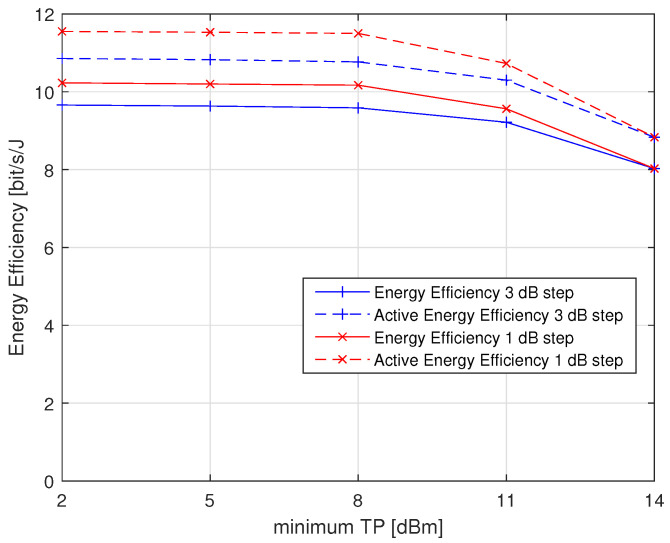
Energy efficiency variation with the minimum TP.

Figure 13 shows that the energy efficiency improvement increases when we decrease the minimum TP used, where we have lower TP levels that can be used in power control. The energy efficiency becomes approximately stable when the minimum TP is below 8 dBm. This can be explained by the fact that the current consumption of the transceiver in transmit mode increases only by 1 mA when the TP increases from 2 to 8 dBm. Moreover, the energy efficiency and the active energy efficiency are better when we decrease the TP step to 1 dB because we will have more discrete TP levels. For example, when the minimum TP is 2 dBm, the improvements in energy efficiency and active energy efficiency using the 1 dB step become 5.9% and 6.3%, respectively, compared to those using the 3 dB step.

#### 6.2.3. Impact of Idle Current

With the technological evolution in the transceiver industry, we expect that we can achieve better energy efficiency and a longer lifetime for nodes by decreasing the idle current. We study the impact of reducing the idle current $I_{id}$ of a LoRa transceiver on the energy efficiency when the number of nodes is 4000 and using $T_{1}$ as the TP set for power control.

Table 13 represents the percentage of energy efficiency improvement when we reduce the idle current. The results show that we achieve a better network efficiency by reducing the idle current. When we reduce the idle current by 16 times, we improve the energy efficiency by 11.6%.

## 7. Conclusions

In this paper, we propose EE-LoRa, an algorithm for SF selection and power control in LoRaWAN networks with multiple gateways. Our algorithm includes two steps, where we first derive the optimal SF that maximizes the network’s energy efficiency, defined as the ratio between the throughput and energy consumption. We make use of the Dinkelbach method to transform the fractional problem into a parameterized one. We solve this problem using the fmicon solver after ensuring that we work in the convexity domain. After finding the optimal node distribution among different SFs, we use the adaptive SNR threshold method to deduce the new SF at each node. In the second stage, we apply a power control to configure the TPs. The simulation results showed that EE-LoRa enhances the energy efficiency of LoRaWAN networks compared to legacy LoRaWAN and relevant state-of-the-art algorithms.

## Figures and Tables

**Table 1:** Summary of relevant proposed algorithms.

Reference	Objective	SF Selection	Power Control	Multiple Gateways
[12]	maximize the minimum packet reception probability for all nodes	✓		
[13]	maximize network throughput	✓		✓
[14]	reduce the pressure that some nodes put on some gateways	✓		✓
[15]	achieve equal collision probability among different SFs	✓	✓	
[16]	improve reliability	✓	✓	✓
[17]	reduce collisions and energy consumption	✓		
[18]	reduce the packet error rate	✓		✓
[19]	improve the PDR and reduce energy consumption	✓	✓	✓
EE-LoRa	maximize energy efficiency	✓	✓	✓

**Table 3:** ToA for different SFs, when payload length = 50 bytes, BW=125 kHz, coding rate = 4/5.

SF	7	8	9	10	11	12
ToA [s]	0.1245	0.2097	0.3801	0.6816	1.206	2.254

**Table 4:** SNR thresholds of different SFs for 868 MHz band, bandwidth 125 kHz [21].

SF	7	8	9	10	11	12
Required SNR [dB]	−7.5	−10	−12.5	−15	−17.5	−20

**Table 5:** Example of node distribution at different SF when $N_{t}$ = 500 nodes.

*s*	7	8	9	10	11	12
D(s)	474	15	4	4	2	1
N(s)	500	26	11	7	3	1

**Table 6:** System model parameters.

Parameters	Description
λ	Packet generation rate
*T*	Time between two successive transmissions
ToA(s)	Time to transmit a packet using SF *s*
$T_{rx1}\left(s\right)$	First receive window duration on SF *s*
$T_{rx2}\left(s\right)$	Second receive window duration on SF *s*
R_D1	RECEIVE_DELAY1 Time from transmitting before opening the first receive window
R_D2	RECEIVE_DELAY2 Time from transmitting before opening the second receive window
*k*	Payload size
*d*	Duty cycle
$N_{t}$	Number of nodes
D(s)	Number of nodes that use SF *s*
N(s)	Number of nodes that use SF ≥ *s*
p(s)	Ratio of nodes that use SF *s*
*l*	Transmission power level in dBm
$I_{tx}\left(l\right)$	Current consumption in transmit mode at transmission power level *l*
$I_{rx}$	Current consumption in receive mode
$I_{st}$	Current consumption in standby mode
$I_{id}$	Current consumption in idle mode
*V*	Node operating voltage
$d_{1}$	Probability that a node successfully receives a downlink message at RX1
$d_{2}$	Probability that a node does not successfully receive a downlink message at RX1

**Table 7:** Maximum number of nodes at each SF, λ = 6 packets/h, *k* = 40 bytes.

*s*	7	8	9	10	11	12
D(s)	5612	3264	1831	1043	561	303

**Table 8:** Current consumption at each TP.

*l* (dBm)	2	3	4	5	6	7	8	9	10	11	12	13	14
$I_{tx}$ (mA)	24	24	24	25	25	25	25	26	31	32	34	35	44

**Table 9:** TP set description.

Set	Description
$T_{1}$	3 dB step
$T_{2}$	1 dB step

**Table 10:** ToA and λ for different SFs, *k* = 40 bytes.

SF	ToA [s]	λ [Packets/h]
7	0.1048	343.32
8	0.1802	199.75
9	0.3211	112.1
10	0.5636	63.87
11	1.0485	34.33
12	1.9398	18.55

**Table 11:** Simulation parameters.

Parameter	Value
Number of nodes $N_{t}$	[1000–4000]
Number of gateways *r*	4
Network Layout	Square, 7 km side
Path loss model	Okumura–Hata urban and dense urban
Spreading Factor SF	s∈Ω={7,8,9,10,11,12}
Transmission Power TP level	l∈ [2 dBm, 14 dBm]
Carrier Frequency	868 MHz
Bandwidth	125 kHz
Coding Rate	4/5
ED/GW antenna	3 dBi omnidirectional
Gateway height	30 m
Node height	1.5 m
Packet generation rate λ	6 packets/h
Payload size *k*	40 bytes
Preamble length $n_{pream}$	8 bits
*T*	720 s
R_D1	1 s
R_D2	2 s
*V*	3.3 V
$I_{rx}$	10.5 mA
$I_{st}$	1.4 mA
$I_{id}$	1.5 μA
Node battery capacity *C*	1800 mA.h

**Table 12:** Example of node distribution at different SFs when $N_{t}$ = 4000 nodes.

*s*	7	8	9	10	11	12
D(s)	3773	126	62	28	8	3

**Table 13:** Energy efficiency improvement by reducing the idle current when $N_{t}$ = 4000 nodes.

Idle Current	$I_{\mathrm{id}}/2$	$I_{\mathrm{id}}/4$	$I_{\mathrm{id}}/8$	$I_{\mathrm{id}}/16$
% of energy efficiency improvement	5.16	9.05	10.99	11.6

## Data Availability

The data used to support the reported results of this paper are available upon request.
